# Effect of patient-centered and family-centered self-care education program on the quality of life of patients with multiple sclerosis: a quasi-experimental study

**DOI:** 10.1186/s12912-023-01492-6

**Published:** 2023-10-18

**Authors:** Zahra Rooddehghan, Mozhgan Moghaddasi Nezhad, Masoumeh Zakerimoghadam, Raoofeh Karimi

**Affiliations:** School of Nursing and Midwifery, Tehran University of Medical Sciences, Nosrat St. Tohid Sq, Tehran, 141973317 Iran

**Keywords:** Multiple sclerosis, Self-care, Patient-centered, Family-centered, Care, Quality of life, Fatigue

## Abstract

**Background:**

Performing self-care behaviors education improves the quality of life of MS patients and reduces their fatigue. This study was conducted with the aim of comparing the effect of patient-centered and family-centered self-care training programs on the quality of life and fatigue of patients with multiple sclerosis.

**Methods:**

This is a quasi-experimental study that was conducted on the MS patients referred to the Iranian MS Association. Sampling was done by convenience method from November 2017 to September 2018. To create a random sequence in the three groups, blocks of nine were used. The control group received no intervention but the intervention groups 1 and 2 received the desired training in the form of workshop with the difference that in the intervention group 2, the patient participated in the training sessions along with one of his/her family members. The phone call follow-up was continued for 8 weeks after the last session. The questionnaire of quality of life in patients with MS (MSQOL-54), fatigue scale (FIS) and demographic information form were used for data collection. The collected data was analyzed by SPSS-16 statistical software, using descriptive (Mean and Standard deviation) and statistical statistics (paired t-test, Analysis of variance and Bonferroni).

**Result:**

A statistically significant difference in the mean scores of quality of life(53/16 ± 15/19 vs 56/03 ± 14/40 vs 52/48 ± 21/20)(*P* < 0.001) and fatigue(50/08 ± 3/28 vs 46/54 ± 28/69 vs 56/11 ± 27/93) (*P* < 0.001) was observed between both patient-centered and family-centered groups and the control group.

**Conclusions:**

Considering the importance and role of the family and nurses in the care and education of patients with multiple sclerosis, it is possible to improve the quality of life and reduce their fatigue by providing self-care training packages to patients and their families.

## Background

MS is a chronic autoimmune disease that is the main cause of non-traumatic neurological disability and affects the central nervous system and the optic nerve. MS attacks myelinated axons and destroys them to varying degrees. MS courses are variable and unpredictable [1–3]. The cause of MS is unknown, but genetic, environmental, infectious, and dietary factors have been introduced as contributors of MS [4, 5]. This disease manifests itself with different symptoms such as fatigue, visual impairment, muscle weakness, bladder and excretion dysfunction, sexual dysfunction, balance disorder, cognitive decline, and emotional/mental disorders [6].

According to statistics, MS is one of the most common chronic disease in Iran and in the world [3]. Approximately 2.3 million people worldwide have MS, which is most prevalent in North America, Western Europe, and Australia [7]. The prevalence of this disease in Iran is 3.29 per 100,000 and its incidence is 3.4 per 100,000 people and the incidence in Iranian women is more than 3 times that of men. The incidence of MS in Iran reached its highest level in 2014 (1.42 per 100,000 people) and then decreased so that it is now stable [8]. It usually affects young patients between 20–40 years old [8, 9]. This disease also affects different aspects of one’s quality of life [10].

Today, several definitions have been proposed for the quality of life. Quality of life is a dynamic and subjective structure that focuses on comparing the past life situation with recent events in all positive and negative aspects. The subjective nature of the quality of life is concerned with people's perception of their life situation instead of the reports of others, and it originates from the satisfaction or lack of satisfaction with the areas of life that are important to the individual [11]. According to the definition of World Health Organization, quality of life is a personal understanding of one’s place in life in the context of culture and value system, which is related to goals, expectations, standards and concerns [12]. Studies show that patients with MS have a lower quality of life than healthy people [13].

Fatigue is one of the main factors that reduces quality of life in MS. Fatigue refers to the lack of physical and mental energy in daily living activities, which is associated with depression, sleep disorder and pain in patients with MS that are aggravated during the treatment process [14]. Fatigue has been reported in 50–90% of patients with MS (14–40%). Fatigue can have a negative impact on the different aspects of life and daily activities and work [3, 15]. It also has a negative impact on the patient’s quality of life [16]. Unfortunately, there is no cure for fatigue caused by MS, and drug treatments are only partially effective [17].

Thus, training can be somewhat effective in controlling fatigue. According to Orem's definition, one of the ways to improve the quality of life in chronic diseases is to teach and help patients to perform self-care behaviors. This training in chronic patients ultimately improves their quality of life. Self-care is a learned regulatory function in humans, which is based on the ability of people to perform self-care activities [18]. Barnsteine ​​et al. [19] states that, in the United States, two types of patient-centered and family-centered care have been proposed due to social pressures and factors influencing health.

Patient-centered care refers to a care that is provided based on respect, and responds to the patient's personal needs [19, 20]. In contrast, family-centered care is a philosophy of care that can replace patient-centered care [21]. Family-centered care refers to a care that is more appropriate, safer and more specific, and involves patient and his/her family in the care process [20]. The purpose of this care is to create a relationship between patients, families and health care providers, and to improve the abilities of family members in certain areas to overcome obstacles to health and well-being [22, 23]. Considering that the disease process in MS ranges from complete patient independence to total reliance of patient on his/her family, paying attention to both types of patient-centered and family-centered care seems necessary.

Considering that the process of MS disease includes complete independence of the individual in self-care to dependence on the family for care and the role of nurses in the care and education of these patients and their families, and the time that nurses spend with families, therefore it is necessary to pay attention to both types of patient-centered and family-centered care in these patients. Since the results of the literature search show that despite the existence of evidence of the importance of family support in improving the health, wellness and self-care of these patients, the role of the family has been addressed in fewer studies, and so far there has been no comparison between the effect of patient-centered and family-centered self-care education on the quality of life and fatigue of patients with MS. Also, in the field of family-centered self-care educational program, the effect of the family-centered empowerment model on the knowledge, performance, and attitude of patients with MS, not the quality of life and fatigue, has been investigated. The present study was conducted with the aim of comparing the effect of patient-centered and family-centered self-care educational program on the quality of life and fatigue of patients with multiple sclerosis.

## Method

### Study design and population

This is a quasi-experimental study that was conducted on 228 patients with MS referred to the Iranian MS Association in Tehran. This center is a charity, where people with MS attend to receive health care services. This study was carried out from November 2017 to September 2018.

### Sampling procedure

The required sample size was calculated at the confidence level of 95% and the power of 80%, and due to the fact that the list of patients with MS in the last one year was not available to the researcher, the convenience method was done for sampling until reaching the desired number of samples in each group. The research samples were selected based on the inclusion criteria from the research population, which included all MS patients referred to the Iranian MS Association. After the researcher contacted the patients who met the inclusion criteria, they were invited to participate in the study and the necessary explanations were given to them. If they agreed to participate in the study, they were asked to attend the MS Association and a written consent form was obtained from them, and the necessary arrangements were made with them regarding the time of the meetings in the MS Association.

### Inclusion and exclusion criteria

Entry criteria for patients included; having definite diagnosis of MS based on clinical findings, laboratory tests and specialist’s diagnosis, not being in the acute stage of the disease, not participating in an educational or research program based on educational intervention related to MS, having the ability to read and write, and being in the age range of 18 to 65 years. The inclusion criteria for the intervention group 2 also included; being a first-degree family member of the patient, being active and interested in patient care, being 18 years old or older, living with the patient, being literate and not having MS. Entering the acute phase of the disease, non-participation in telephone follow-up, non-participation of the family in the intervention group 2 in the training sessions, declaration of unwillingness to participate in the study were considered as exclusion criteria in this study.

### Randomization

A limited randomization method (block randomization) was used to randomly assign the samples to one of the three study groups (intervention 1, intervention 2, and control). In the block randomization, equal blocks of 9 were used, and in order to hide the random allocation, the method of sealed envelopes with random sequence was used. For this purpose, a number of envelopes were prepared and each of the random sequences was written on a card and the cards were placed inside the envelopes. To maintain the random sequence, the outer surface of the envelopes was numbered in the same order. Finally, the lids of the envelopes were glued and then, they were placed inside a box in the same way. According to the order by which, the participants entered the study, one of the envelopes was opened and the assigned group of that participant was revealed. The English letter A was the symbol of control group, the letter B was the symbol of intervention group 1 (patient-centered training), and the letter C was the symbol of intervention group 2 (family-centered training).

### Study instruments

1. The researcher-made demographic information form: This questionnaire included 10 questions related to demographic information (age, gender, height, weight, marital status, level of education, number of children, occupation, income level, type of financial support) and 6 questions related to the disease information (the duration of disease, number of relapses in the last year, number of hospitalizations in the last year, first symptom of the disease, the most important debilitating problem from the patient's point of view, and having a disease other than MS).
2. Quality of life questionnaire (MSQOL-54): This questionnaire was developed in 1990 by Vickrey et al. for the first time, which is the most famous tool to measure quality of life. This questionnaire was developed initially in English, which was later translated into different languages [24]. The MSQoL-54 is known as a health-related self-report questionnaire containing 54 items, categorized into 12 sub-scales: physical health, role limitations-physical, emotional well-being, pain, energy, health perceptions, social function, cognitive function, health distress, sexual function, change in health, and overall QoL [25]. The minimum and maximum score of the quality of life and its dimensions in this questionnaire are in the range of 0 to 100, where a higher score indicates a higher quality of life [26]. In the original version, Cronbach's alphas for each dimension range from 0.75 to 0.96. And the alpha for Mental and Physical QoL was 0.81 and 0.88 [27]. The reliability of the Persian version of this questionnaire was conducted in 2016 by Qaim et al. on patients with MS and its value was reported as 0.962 [28]. Kazem Mohammad et al. and Sangalji et al. have reported the internal consistency of 0.86 [29, 30] and Cronbach's alpha of 0.88 for this questionnaire [15].
3. Fatigue Impact Scale (FIS): The fatigue impact scale was first used by Fisk in 1994 to evaluate the impact of fatigue on daily life activities. This scale includes 40 questions that evaluate the limit of people's performance in 3 dimensions, including cognitive dimension (in 10 questions related to concentration, memory, thinking and organization of thoughts), physical dimension (10 questions related to motivation, effort, tolerance and coordination) and social dimension (20 questions related to the effect of fatigue on isolation, emotions, work pressure and undertaking tasks). The impact of fatigue on these dimensions is scored on a 5-point Likert scale that ranges from 0 (no problem) to 4 (severe problem) [31]. The intraclass correlation (ICC) values for interrater reliability on the cognitive subscale, social subscale, physical subscale, and total score were 0.86, 0.95, 0.89, and 0.98, respectively. In addition, the test–retest reliability values were equal to 0.78, 0.92, 0.86, and 0.93, respectively [32]. Heidari et al. in order to investigate the validity and reliability of the Persian version of (FIS) among MS patients in Iran, reported the content validity index of 0.85 for the whole scale, as well as its internal consistency with Cronbach's alpha coefficient of 0.953 [33]. The Cronbach’s alpha of the FIS was 0.95, which indicates the high reliability of the FIS [34].

All the data collection tools along with the objectives and method of the study were provided to ten members of the Faculty of Nursing and Midwifery of Tehran University of Medical Sciences and they confirmed the face and content validity according to the objectives of the study.

### Intervention

Necessary explanations were given to the president and officials of MS Association and a research membership card was received from the association, and then sampling started. The educational content was prepared in the form of a booklet using library resources and reliable domestic and foreign websites. The booklet was then given to 5 respected professors at the Faculty of Nursing and Midwifery of Tehran University of Medical Sciences to confirm its reliability. PowerPoint was also prepared for presentation in training sessions. At first, the study objectives were explained to the patients, and then they were allocated in three groups of intervention 1 (patient-centered training), intervention 2 (family-centered training) and control by random block allocation method (blocks of 9). The control group only received the routine care of the association. The association did not have any routine educational training for patients, and only offered classes such as yoga, music, chess, etc. The educational contents of training were presented to the intervention groups 1 and 2 in a form of educational booklet, with the difference that in the intervention group 2, the patient participated in the training sessions along with one of his/her family members (a person who had an impact on the patient from the family), while patients in the intervention group 1 participated in the training sessions on their own.

Training sessions were held in the form of workshops with 8 to 10 people, and they were repeated until reaching the desired sample size (47 people). Each workshop was held on two consecutive days and each day in two one-hour sessions with a 15-min break between each session. The days of workshops were coordinated in advance with the patients. The content of training sessions was presented in the same way for both intervention groups 1 and 2 (Table 1).

**Table 1:** The educational content of training sessions presented to the intervention group 1 and 2

Session	Content
1	Getting to know the patients and familiarizing the patients with each other, filling out the questionnaires, explaining the research objectives and methods, getting to know the nature of the disease, its complications, risk factors and its control methods, providing educational booklets to patients after getting to know each other. According to the study objectives, the educational contents were presented through PowerPoint and lectures
2	Familiarizing patients with treatment methods and principles of drug therapy and their side effects
3	Familiarizing patients with diet, appropriate physical activities and stress reduction methods
4	Familiarizing patients with the method of overcoming disease problems, especially fatigue. Familiarizing patients in the intervention group 1 and patients and a family member in the intervention group 2 with the telephone follow-up for 8 weeks after the last training session. Questions and answers, reinforcement of teachings and managing the disease once a week for about 10 min based on the patient's needs

At the beginning of the training sessions, a summary of the previous topics were presented and all questions were answered. After the completion of each workshop, a comprehensive self-care program booklet was provided to patients and families. After the last training session, telephone follow-up was carried out for patients in the intervention group 1 and also the same follow-up was done for patients in the intervention group 2 along with one of their family members for 8 weeks. Telephone calls were made between 8 am and 8 pm on a certain date and time agreed upon by the participants.

Finally, after the completion of the intervention and eight weeks after completing the questionnaires by the participants, the questionnaires were completed again by all three study groups. Patients who were unable or had no time to attend the association received the questionnaire via post and they were asked to send the completed questionnaire through internet messengers. It should be noted that in order to comply with the research ethics, at the end of the study, a copy of the MS self-care training booklet was also provided to patients in the control group.

### Data analysis

After collecting the data, it was entered into SPSS-16 software for analysis, using descriptive statistics (mean, standard deviation) and statistical tests (paired t-test, Analysis of variance and Bonferroni).

### Ethical considerations

Code of ethics (IR.TUMS.FNM.REC.1396.3169) for this study was obtained from the joint ethics committee of the Faculties of Nursing & Midwifery and Rehabilitation of Tehran University of Medical Science. Informed consent was obtained from the participants. The confidentiality and privacy of the information was respected on personal data protection.

## Results

Among 228 patients with MS, 11 were under 20 years old, 2 were over 65 years old, 9 had entered the acute stage of the disease, 26 had already received education related to their disease, and 11 patients or their first-degree family members were not literate (were not able to read and write). Also, 21 patients lived alone and 7 patients lived with their children who were under 18 years of age. All the above-mentioned people were excluded from the study according to the study criteria. It should be noted that in the patient-centered group, one patient did not fill the questionnaires after the intervention and was removed from the study. Finally, patients with multiple sclerosis were divided into three study groups, including patient-centered group (14 men and 32 women, *n* = 46), family-centered group (13 men and 34 women, *n* = 47) and control group (15 men and 32 women, *n* = 47).

The demographic characteristics of the patients are explained in Table 2. There was no statistically significant difference between the three groups in terms of gender (*P* = 0.90) and mean age (*P* = 3.16). Also, patients in the three groups were similar in terms of marital status, education level, number of children, employment status, income level, type of financial support, duration of disease, frequency of disease recurrence, frequency of hospitalization and first symptom of the disease. The results of this study showed a significant difference between the three groups in terms of weakness (*P* = 0.032), fatigue (*P* = 0.001), muscle stiffness (*P* = 0.001), lack of balance (*P* = 0.007) and other problems. However, no significant difference was observed between the three groups in terms of visual impairment (*P* = 0.085), (Table 2).

**Table 2:** Demographic characteristics of the participants in the intervention group 1 (patient-centered), intervention group 2 (family-centered) and control group

Characteristic		Patient-centered (percentage) frequency		Family-centered (percentage) frequency	Control (percentage) frequency	*P*-value
Gender	Male	(30.4) 14		(27.7) 13	(31.9) 15	0.90
	Female	(69.6) 32		(72.3) 34	(68.1) 32	
Age (years)	22–29	(45.65) 21		(48.94) 23	(25.53) 12	3.16
	30–34	(21.74) 10		(31.91) 15	(38.30) 18	
	35–40	(26.09) 12		(12.77) 6	(19.15) 9	
	40 and more	(6.52) 3		(6.38) 3	(17.02) 8	
Marital status	Single	(5) 20		(51.1) 24	(44.7) 21	0.88
	Married	(45.7) 21		(36.2) 17	(40.4) 19	
	Divorced and deceased wife	(10.8) 5		(12.7) 6	(14.9) 7	
Education level	Below high school	(26.1) 12		(25.5) 12	(23.4) 11	0.69
	High school Diploma	(45.7) 21		(34) 16	(36.2) 17	
	University education	(28.3) 13		(40.4) 19	(40.4) 19	
Number of children	One	(26.9) 7		(21.7) 5	(26.9) 7	0.54
	Two	(69.2) 18		(60.9) 14	(57.7) 15	
	Three	(3.8) 1		(17.4) 4	(15.4) 4	
Occupation	Laborer	(2.2) 1		(3.4) 2	(10.6) 5	0.36
	Office worker	(6.5) 3		(12.8) 6	(17) 8	
	Housewife	(34.8) 16		(23.4) 11	(29.8) 14	
	Unemployed	(54.3) 25		(57.3) 27	(42.6) 20	
	Other jobs	(2.2) 1		(2.1) 1	(0) 0	
Income level	It is not enough	(56.5) 26		(48.9) 23	(53.2) 25	0.76
	It is enough to some extent	(43.5) 20		(51.1) 24	(46.8) 22	
Type of support	Insurance	(30.4) 14		(36.2) 17	(36.2) 17	0.81
	Emdad Committee	(2.2) 1		(0) 0	(0) 0	
	Welfare	(4.3) 2		(8.5) 4	(6.4) 3	
	Family	(60.9) 28		(51.1) 24	(57.4) 27	
	Others	(2.2) 1		(4.2) 2	(0) 0	
Duration of disease	5 years and less	(67.4) 31		(76.6) 36	(70.2) 33	0.29
	6–10 years	(23.9) 11		(23.4) 11	(20.5) 12	
	More than 10	(8.9) 4		(0) 0	(4.3) 2	
Number of disease recurrence	No recurrence	(34.8) 16		(23.4) 11	(48.9) 23	0.12
	Once	(43.5) 20		(51.1) 24	(25.5) 12	
	Twice	(15.2) 7		(10.6) 5	(14.9) 7	
	More than twice	(6.5) 3		(14.9) 7	(10.6) 5	
Number of hospitalization	No hospitalization	(28.3) 13		(19.1) 9	(6.4) 3	0.07
	Once	(37) 17		(23.4) 11	(42.6) 20	
	Twice	(19.6) 9		(31.9) 15	(29.8) 14	
	More than twice	(15.2) 7		(25.5) 12	(21.3) 10	
First symptom of the disease	Visual impairment	(67.4) 31		(68.1) 32	(78.7) 37	0.47
	Urinary sphincter disorder	(2.2) 1		(0) 0	(0) 0	
	Motor disorder	(3.4) 2		(6.4) 3	(0) 0	
	Sensory disorder	(26.1) 12		(25.5) 12	(21.3) 10	
Main problem	Fatigue	Yes	(43.5) 20	(38.3) 18	(19.1) 9	0.032
		No	(56.5) 26	(61.7) 29	(80.9) 38	
	Weakness	Yes	(54.3) 25	(34) 16	(17) 8	0.001
		No	(45.7) 21	(66) 31	(83) 39	
	Muscle stiffness	Yes	(8.7) 4	(4.3) 2	(0) 0	0.001
		No	(91.3) 42	(95.7) 45	(100) 47	
	Visual impairment	Yes	(10.9) 5	(8.5) 4	(42.6) 20	0.085
		No	(89.1) 41	(91.5) 43	(42.6) 27	
	Lack of balance	Yes	(45.7) 21	(19.1) 9	(21.3) 10	0.007
		No	(54.3) 25	(80.9) 38	(78.7) 37	
	Others	Yes	(8.7) 5	(0) 0	(0) 0	0.01
		No	(91.3) 41	(100) 47	(100) 47	
Other diseases	Yes	(10.9)		(8.5) 4	(6.4) 3	0.70
	No	(89.1) 41		(91.5) 43	(93.6) 44	
Comorbidity	High blood pressure	Yes	(40) 2	(50) 2	(33.3) 1	0.90
		No	(60) 3	(50) 2	(66.7) 2	
	Diabetes	Yes	(40) 2	(25) 1	(66.7) 2	0.61
		No	(60) 3	(75) 3	(33.3) 1	
	Pneumonia	Yes	(20) 1	(25) 1	(33.3) 1	0.69
		No	(80) 4	(75) 3	(66.7) 2	
	Varicose veins	Yes	(20) 1	(0) 0	(0) 0	0.29
		No	(80) 4	(100) 8	(100) 3	
	Breathing problem	Yes	(0) 0	(25) 1	(100) 3	0.33
		No	(100) 5	(75) 3	(0) 0	

After the 8-week intervention period, Based on the results of analysis of variance, the overall score of quality of life has changed in the patient-centered group + 6.5, in the family-centered group + 9.22, and in the control group + 1.16, and a statistically significant difference was observed in this regard (*P* < 0.001), so that after the intervention, the quality of life in both patient-centered(53/16 ± 15/19) and family- centered(56/03 ± 14/40) groups had improved significantly compared to the control group(52/48 ± 21/20) (Table 3). As the results of Bonferroni test showed, the score of fatigue symptoms was significantly higher in the control group (56/11 ± 27/93) compared to the patient-centered (50/08 ± 3/28) and family-centered (46/54 ± 28/69) groups (*P* < 0.001). Also, the score of fatigue symptoms was significantly higher in the patient-centered group compared to the family-centered group (*P* = 0.007) (Table 4).

**Table 3:** Comparison of the mean scores of quality of life and its dimensions before and after the intervention in the intervention group 1 (patient-centered), intervention group 2 (family-centered) and control group

Quality of life and its dimensions		Pre-intervention	Post-intervention	Statistical tests, paired t-test		
		**(SD) Mean**	**(SD) Mean**	***P***	**df**	**T**
Physical health	Patient-centered	(27.61) 51.74	(24.82) 54.46	0.014	45	2.55-
	Family-centered	(28.13) 60.74	(26.16) 63.94	0.002	46	3.26-
	Control	(25.12) 74.36	(25.53) 73.94	0.68	46	0.40
Physical problems caused by role limitations	Patient-centered	(33.30) 30.43	(26.56) 51.63	< 0.001	45	7.05-
	Family-centered	(33.32) 38.30	(22.10) 46.28	0.079	46	1.79-
	Control	(37.74) 59.04	(38.94) 61.17	0.49	46	0.68-
Emotional problems caused by role limitations	Patient-centered	(38.13) 42.03	(29.49) 62.32	< 0.001	45	4.68-
	Family-centered	(36.54) 39.72	(32.58) 71.63	< 0.001	46	5.25-
	Control	(32.95) 58.16	(36.69) 49.65	0.07	46	1.85
The level of pain	Patient-centered	(21.37) 52.21	(15.68) 63.70	< 0.001	45	7.04-
	Family-centered	(24.47) 59.11	(17.89) 70.28	< 0.001	46	5.64-
	Control	(23.28) 79.96	(23.39) 77.70	0.07	46	1.80
Feeling good	Patient-centered	(5.61) 58.61	(5.57) 59.48	0.22	45	1.21-
	Family-centered	(4.65) 58.72	(3.98) 60.34	0.051	46	2.01-
	Control	(6.74) 65.36	(5.94) 66.13	0.18	46	1.35-
Energy	Patient-centered	(12.64) 40.35	(11.44) 43.39	< 0.001	45	4.61-
	Family-centered	(10.38) 41.70	(8.50) 45.87	< 0.001	46	5.56-
	Control	(10.68) 46.13	(11.92) 43.57	0.003	46	3.14
Understanding of health	Patient-centered	(22.61) 43.80	(18.65) 46.63	0.021	45	2.39-
	Family-centered	(21.89) 39.04	(19.63) 46.38	0.021	46	4.69-
	Control	(25.71) 46.04	(26.40) 44.04	0.23	46	1.21
Social Performance	Patient-centered	(20.35) 51.99	(16.25) 57.25	< 0.001	45	3.95-
	Family-centered	(20.03) 54.43	(14.45) 60.28	< 0.001	46	4.15-
	Control	(18.35) 69.86	(19.22) 67.20	0.01	46	2.70
Malfunction	Patient-centered	(14.88) 56.20	(13.46) 59.24	< 0.001	45	3.80-
	Family-centered	(14.53) 55.53	(12.54) 57.55	0.10	46	1.66-
	Control	(19.68) 73.72	(20.38) 70.32	0.001	46	4.01
Stressful factors	Patient-centered	(21.36) 52.17	(18.00) 55.98	0.001	45	3.56-
	Family-centered	(17.27) 50.32	(13.67) 56.49	0.001	46	4.53-
	Control	(21.43) 61.28	(25.14) 57.77	0.058	46	1.94
Women's sexual performance	Patient-centered	(26.45) 44.05	(23.08) 45.83	0.27	13	1.14-
	Family-centered	(30.55) 33.33	(22.18) 43.75	0.10	7	1.85-
	Control	(19.88) 76.94	(16.11) 74.38	0.33	12	0.99
Men’s sexual performance	Patient-centered	(30.69) 32.28	(30.22) 33.32	0.35	7	1.00-
	Family-centered	(30.47) 32.41	(26.84) 40.74	0.02	8	2.68-
	Control	(31.88) 54.76	(34.32) 54.76	-	-	-
Health related changes	Patient-centered	(20.15) 45.10	(20.15) 45.10	-	-	-
	Family-centered	(17.91) 38.83	(17.86) 39.36	0.32	46	1.00-
	Control	(19.09) 48.40	(20.07) 47.87	0.56	46	0.57
Classification of sexual function	Patient-centered	(25.50) 30.68	(25.27) 36.36	0.057	22	8.29
	Family-centered	(28.27) 29.41	(21.86) 38.23	0.08	17	1.85-
	Control	(24.19) 52.50	(22.94) 50.0	0.33	19	1.00
Overall assessment of quality of life	Patient-centered	(17.87) 47.10	(15.19) 53.16	< 0.001	45	5.30-
	Family-centered	(19.71) 46.81	(14.40) 56.03	< 0.001	46	7.60-
	Control	(19.95) 51.31	(21.29) 52.48	0.39	46	0.86-

**Table 4:** Comparison of the mean scores of fatigue before and after the intervention in the intervention group 1 (patient-centered), intervention group 2 (family-centered) and control group

Level of fatigue and its dimensions		Pre-test	Post-test	Statistical tests, paired t-test And The result of analysis of variance				The result of analysis of variance Pre-test	The result of analysis of variance Post-test
		**(SD) Mean**	**(SD) Mean**	**F**	***P***	**df**	**T**		
Cognitive (0–40)	Patient-centered	(6.92) 15	(7.30) 11.27		0.002	45	3.66	F = 27.55 *P* < 0.001	F = 8.38 *P* < 0.001
	Family-centered	(6.40) 14.47	(6.29) 10.55	4.12	< 0.001	-	-		
	Control	(5.47) 6.38	(6.32) 12.43	8.29	0.20	45			
Physical (0–40)	Patient-centered	(8.44) 20.50	(15.33) 14.62		< 0.001	45	5.54	F = 36.45 *P* < 0.001	F = 18.34 *P* < 0.001
	Family-centered	(7.83) 19.70	(14.32) 13.30	5.96	< 0.001	-	-		
	Control	(7.83) 7.83	(14.36) 16.40	8.38	0.57	45			
Social (0–80)	Patient-centered	(15.87) 31.72	(8.48) 24.50		< 0.001	45	6.63	F = 22.45 *P* < 0.001	F = 12.34 *P* < 0.001
	Family-centered	(14.82) 32.55	(7.48) 22.97	7.57	< 0.001	-	-		
	Control	(39/12) 91/14	(51/7) 81/26	3.66	0.01	45			
Fatigue (0–160)	Patient-centered	(3.25) 67.21	(3.28) 50.08		< 0.001	45	9.59	F = 28.37 *P* < 0.001	F = 27.10 *P* < 0.001
	Family-centered	(28.64) 66.72	(28.69) 46.54	7.47	< 0.001	-	-		
	Control	(25.10) 29.12	(27.93) 56.11	18.28	0.08	45			

## Discussion

The present study was conducted with the aim of comparing the effect of patient-centered and family-centered self-care educational programs on the quality of life and fatigue of patients with multiple sclerosis. The findings of present study showed a statistically significant difference between the intervention groups 1 and 2 in terms of the mean scores of quality of life and fatigue compared to the control group. It means that after the intervention, the quality of life of patients in the family-centered group improved significantly compared to the patient-centered groups, and also the fatigue of patients in the family-centered group significantly reduced compared to the patient-centered.

In a semi-experimental study, Sahib al-Zamani et al. [35] conducted a self-care training among 60 patients with MS and the results showed that self-care training improved all areas of quality of life, and the differences in both physical and mental areas and overall quality of life score were significant (*P* < 0.001), which is in line with the present study [35]. The findings of present study are also in line with the results of a study by Parsa et al. [36] who examined the effectiveness of patient-centered educational and therapeutic intervention on improving the quality of life of patients with multiple sclerosis. In their study, the findings showed that educational interventions were effective in improving health status, reducing burnout severity and pain, and promoting mental health and subject perception [36]. According to the studies conducted on many chronic diseases and due to the vital role that the family plays in the care of patients, the use of family-centered care is considered more logical than patient-centered care. This issue has been proven in many studies. In this regard, the results of Mousai et al.’s [37] study showed that the intervention based on the family-centered empowerment model increased the self-care behaviors of MS patients. The quality of life of these patients can be improved with the participation of their families in health education [37]. Based on the findings of Homayuni et al.'s [38] research, paying attention to factors such as family and holding family therapy sessions and providing support systems can help to improve the quality of life of these patients [38]. In addition, the results of another study by Khorrami and colleagues [39], who evaluated the effectiveness of family-centered training program on caregivers, showed that family-centered education (with any method) can improve the quality of life of patients and increase the knowledge of caregivers [39].

Very few studies have compared the effectiveness of patient-centered and family-centered self-care training program in patients with MS, and the results of these studies are different according to the research population, the demographic characteristics of the patients, the degree of disability, and other environmental factors. However, a comparative study on patients with Myocardial infarction showed that family-centered self-care training was more effective in reducing heart rate irregularities than patient-centered self-care training [40]. In type II diabetic patients, family-centered self-care educational interventions have been shown to be more effective than patient-centered interventions in improving the quality of life of patients [41]. In a clinical trial conducted on dialysis patients, the results of this study showed that family-centered education was more effective on the patient's adherence to treatment regimen than patient-centered education. Therefore, they recommended family-centered educational interventions to be prioritized in dialysis patients [42].

Very few studies have investigated educational interventions to reduce fatigue in MS patients. Meanwhile, studies conducted on symptoms reduction and severity of fatigue have mostly emphasized on sport exercises and cognitive-behavioral interventions [43]. For example, Ebrahimi Atri et al. [44] in a clinical trial study, subjected 20 women with MS to patient-centered self-care training by performing endurance exercises. The results of this study showed a significant difference in the mean scores of fatigue intensity and patient balance before and after the training in both groups. This study proposed non-pharmacological treatments to control the symptoms of MS, and also referred to resistance and endurance exercises as an effective factor in reducing the intensity of fatigue and balance of people with MS [44]. The results of this research are consistent with the findings of present study, as they both indicate the effectiveness of self-care educational interventions in MS patients and patients with other chronic diseases. Studies show that a multidisciplinary management and drug and non-drug treatments are needed to manage fatigue in these patients which includes rehabilitation treatments including exercise and strengthening, water treatments, and behavioral, educational and drug interventions.

Due to the physical limitations they experience as a result of the disease, MS patients are often disturbed in their emotional reactions and mental state, so the support of family and relatives has a considerable impact on increasing their morale and life expectancy. The findings of above-mentioned studies, which also prove the superiority of family-centered educational interventions over patient-centered intervention in improving the quality of life of people with chronic diseases, are in line with the results of present study.

People's lack of motivation to participate in training classes, the mental state of some participants at the time of training sessions, and the indifference of some participants towards improving their health status were among the limitations of this study. The researcher tried to control these variables as much as possible by holding an interactive training session and encouraging patients in both groups as well as the families of patients in the family-centered group. In addition, due to the fact that the list of clients for the last year was not available at the association, the convenience sampling method was used for sampling.

It is suggested to conduct more studies on the topics of: investigating other aspects of the effectiveness of patient-centered and family-centered self-care education interventions in these patients, comparative studies between these two educational methods in other chronic diseases, and conducting qualitative studies in family-centered care in MS patients.

## Conclusion

Results of this study showed that the use of patient-centered and family-centered educational interventions is effective in patients with multiple sclerosis. Also, due to the importance of family's role in the care of MS patients, the use of family-centered care seems more logical than patient-centered care. Family-centered care, by involving both the patient and the family in care process, creates a cooperation between family and patients, provides physical comfort and emotional support to the patient and his/her family, improves the attitude towards the patient's concerns and cultural beliefs, and finally provides a dedicated care for patients. Therefore, family-centered is preferable to patient-centered care. By providing brochures and self-care training packages to patients and their families, and also holding self-care training courses in MS treatment centers, it is possible to improve the quality of life of MS patients and reduce their fatigue.

## Data Availability

The datasets used and/or analyzed during the current study are available from the corresponding author on reasonable request.
